# MALAT1-related signaling pathways in colorectal cancer

**DOI:** 10.1186/s12935-022-02540-y

**Published:** 2022-03-19

**Authors:** Wen-Wen Xu, Jin Jin, Xiao-yu Wu, Qing-Ling Ren, Maryam Farzaneh

**Affiliations:** 1 Department of Gynaecology, The Affiliated Hospital of Nanjing University of Chinese Medicine, Nanjing , 210029 Jiangsu China; 2 Department of Surgical Oncology, The Affiliated Hospital of Nanjing University of Chinese Medicine, Nanjing, 210029 Jiangsu China; 3 Fertility, Infertility and Perinatology Research Center, Ahvaz Jundishapur University of Medical Sciences, Ahvaz, Iran

**Keywords:** Colorectal cancer, Long non-coding RNAs, MALAT1, Signaling pathways, miRNAs

## Abstract

Colorectal cancer (CRC) is one of the most lethal and prevalent solid malignancies worldwide. There is a great need of accelerating the development and diagnosis of CRC. Long noncoding RNAs (lncRNA) as transcribed RNA molecules play an important role in every level of gene expression. Metastasis‐associated lung adenocarcinoma transcript‐1 (MALAT1) is a highly conserved nucleus-restricted lncRNA that regulates genes at the transcriptional and post-transcriptional levels. High expression of MALAT1 is closely related to numerous human cancers. It is generally believed that MALAT1 expression is associated with CRC cell proliferation, tumorigenicity, and metastasis. MALAT1 by targeting multiple signaling pathways and microRNAs (miRNAs) plays a pivotal role in CRC pathogenesis. Therefore, MALAT1 can be a potent gene for cancer prediction and diagnosis. In this review, we will demonstrate signaling pathways associated with MALAT1 in CRC.

## Introduction

Colorectal cancer (CRC) or colorectal adenocarcinoma is a complex and the third cause of malignancies in the world [1, 2]. CRC usually arises from the hyper-proliferative glandular and epithelial cells in the large intestine [3]. Several environmental and genetic factors can stimulate the accumulation of mutations and oncogenes, and inhibit tumor suppressor genes in colon epithelial cells [4]. Currently, surgical resection [5], chemotherapy [6], and radiotherapy [7] are the common types of treatments for CRC [8]. Recently, molecular targeted therapy has emerged as a novel treatment option for targeting CRC cells [9, 10]. Some studies provided evidence that cancer-specific long non-coding RNAs (lncRNAs) can be utilized for anti-CRC drugs [11, 12]. LncRNA (> 200 nucleotides in length) are transcribed RNA molecules that directly or indirectly regulate a variety of biological functions [13]. It has been shown that many lncRNAs are involved in human diseases and cancers through the induction of cell cycle progression, invasion, and metastasis [14]. Metastasis‐associated lung adenocarcinoma transcript‐1 (MALAT1) is a conserved and well-characterized lncRNA that plays an important role in various biological processes through diverse mechanisms [15]. Under hypoxia conditions, MALAT1 plays an important role in inflammation, angiogenesis, and metastasis [16].

The expression of MALAT1 was first detected in patients with non-small cell lung cancer (NSCLC) [17, 18]. The expression of MALAT1 has been upregulated in multiple cancer types include liver [19], cervix [20], breast [21], colorectal [22], renal [23], prostate [24], gastric [25] and other cancers [26, 27]. In tumor cells, MALAT1 by targeting several transcription factors, growth factors, hormones, and epigenetic histone modifications can mediate cancer cell proliferation, tumorigenicity, autophagy, epithelial-mesenchymal transition (EMT), metastasis, and drug resistance phenotypes [28–31]. Recent studies elucidated the role of MALAT1 in CRC cell growth, migration, invasion, and metastasis [32, 33]. MALAT1 was reported to target several CRC-related pathways such as Wnt/β-catenin, YAP, SOX9, RUNX2, Snail, EGF, PI3K/AKT/mTOR, P53, and VEGF [34, 35]. Besides, MALAT1 by suppressing multiple microRNAs (miRNAs) plays a pivotal role in CRC pathogenesis [36, 37]. miRNAs are epigenetic modulators that target mRNAs and function in various biological and pathological processes [38].

Therefore, MALAT1 can be a potent biomarker for CRC prediction and diagnosis [39, 40]. In this review, we summarized MALAT1-related signaling pathways in CRC.

## Biogenesis of MALAT1

**Fig. 1:**
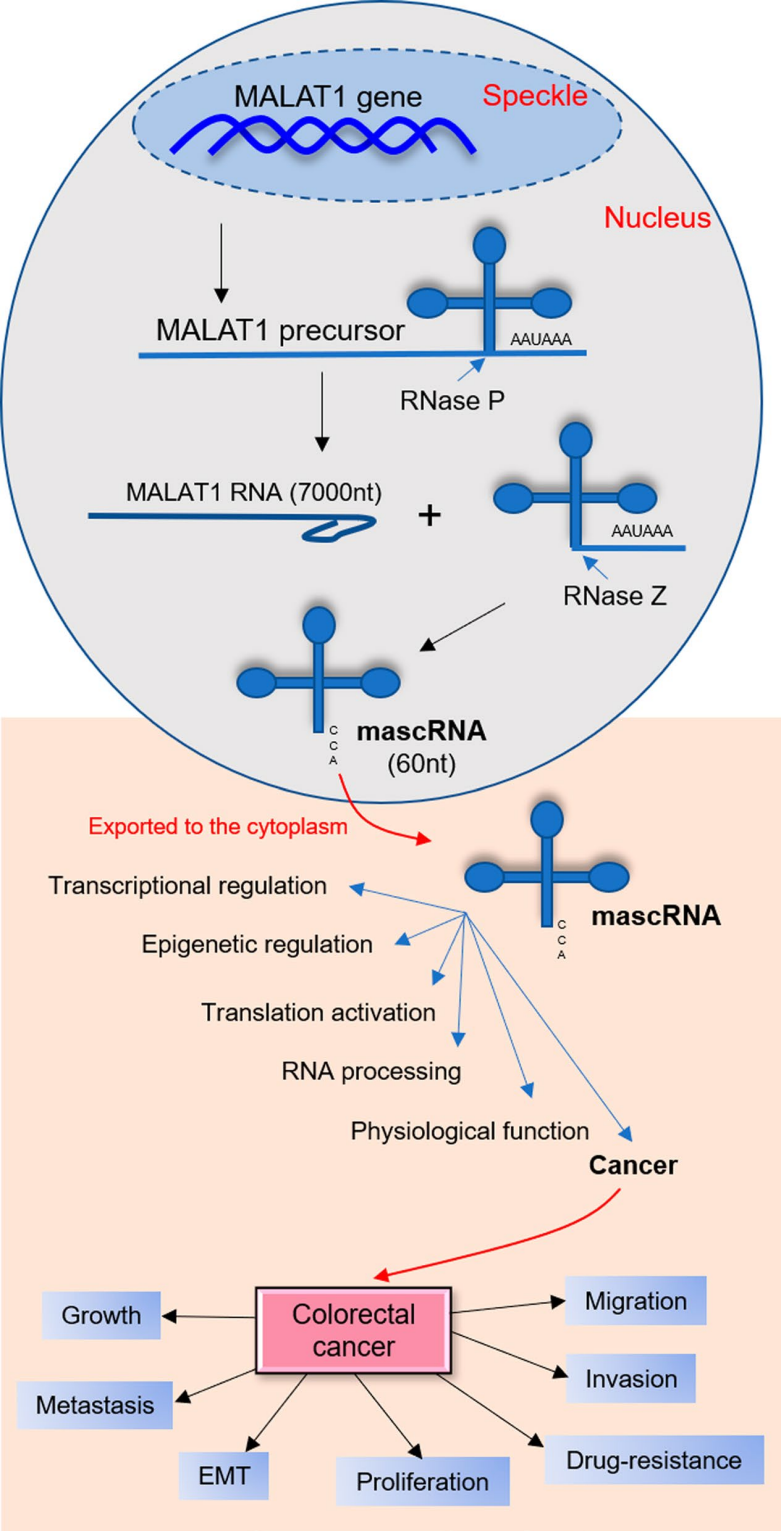
Characterization of MALAT1. MALAT1 contains a highly conserved triple-helix element at the 3′ end named MALAT1-associated small cytoplasmic RNA (mascRNA) that protects the 3′ end from degradation and facilitates the localization of MALAT1. mascRNA is a tRNA-like structure that is separated from MALAT precursor by tRNA endonucleases RNase P. Then, pre-mature MALAT1 with a short poly(A) tail-like moiety is cleaved by tRNA endonucleases RNase Z. MALAT1 plays functional roles in transcriptional regulation, translation activation, epigenetic regulation, RNA processing, physiological processes, and cancer

MALAT1 (known as nuclear-enriched abundant transcript 2 (NEAT2) or hepcarcin (HCN)) is the most widely investigated lncRNA and RNA polymerase II transcripts that localizes to nuclear speckles [41, 42]. MALAT1 coding gene is located on human chromosome 11q13.1 (> 8000 nucleotides) [28] and functions in alternative splicing [26]. The MALAT1 precursor contains a highly conserved triple-helix element at the 3′ end named MALAT1-associated small cytoplasmic RNA (mascRNA) that protects the 3′ end from degradation and facilitates the localization of MALAT1 [43, 44]. mascRNA with a tRNA-like structure is separated from MALAT precursor by tRNA endonucleases RNase P to generate pre-mature MALAT1 [45, 46]. RNase P can also generate a 61-nt tRNA-like small RNA at the 5’ end of the abundant MALAT1 transcript which is exported to the cytoplasm [47]. Pre-mature MALAT1 with a short poly(A) tail-like moiety is cleaved by tRNA endonucleases RNase Z in the nucleus, prior to addition of the CCA motif. After processing, mascRNA with CCA trinucleotide tail exported to the cytoplasm, while the stable MALAT1 transcript accumulates in the nucleus [48] (Fig. 1).

MALAT1 interacts with multiple miRNAs and small nuclear RNAs (snRNAs) to regulate various biological processes in human tissues [46]. It has been reported that several nuclear speckles such as RNPS1 (RNA-binding protein with serine-rich domain 1), SRm160 (serine/arginine-rich (SR)-related protein), and IBP160 (intron binding protein) regulate the proper localization of MALAT1 to nuclear speckles [17]. MALAT1 also changes the distribution of SR splicing factor (SRSF), SON1, hnRNPC, and hnRNPH1 as pre-mRNA splicing factors [17, 49]. MALAT1 can target polycomb repressive complex 2 (PRC2) components (enhancer of Zeste 2 (EZH2), SUZ12, and EED), increase trimethylation of histone H3 at lysine 27 (H3K27me3), and decrease target gene or miRNA expression [50]. Down-regulation of MALAT1 influenced the distribution of SR proteins and changed splicing of pre-mRNA [51]. Nowadays, various specific gene manipulation methods using siRNAs, miRNAs, and shRNA mediated the knockdown of MALAT1 have been introduced for diagnostic, prognostic, and therapeutic values of MALAT1 and its downstream targets [17, 52]. Although the exact mechanism of MALAT1 is unclear, its expression is misregulated in numerous human malignancies. MALAT1 as a competing endogenous RNA (ceRNA) can sponge miRNAs to inhibit their expression and stimulate their downstream targets.

MALAT1 was suggested to be a prognostic marker in multiple cancerous tissues. Below, we summarized the overview function of MALAT1 in colorectal cancer.

## The role of MALAT1 in colorectal cancer

**Fig. 2:**
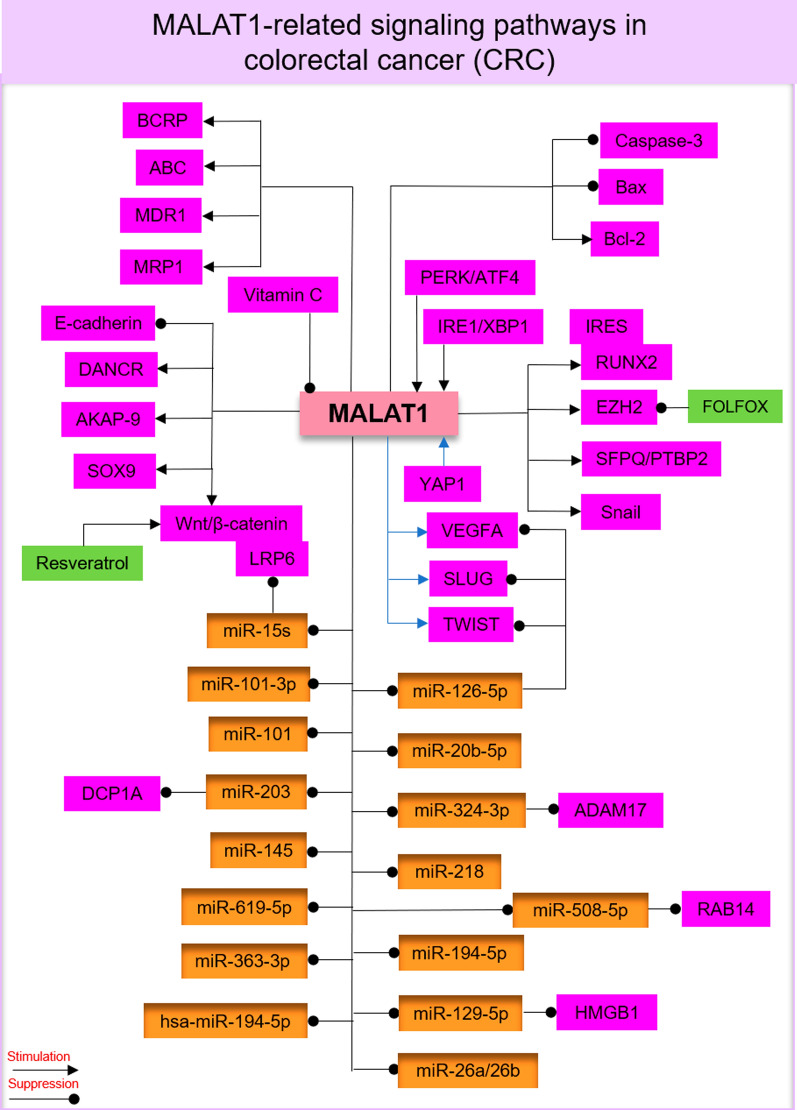
MALAT1-related signaling pathways in CRC. MALAT1 by targeting multiple signaling pathways and microRNAs (miRNAs) plays a pivotal role in CRC pathogenesis

The MALAT1 fragment at the 3' end is known to be associated with CRC cell metastasis [47, 53]. However, the exact mechanisms of MALAT1 in CRC are not fully understood. Previous studies have established that MALAT1 promotes CRC cell proliferation, apoptosis, migration, metastasis, and angiogenesis (Table 1). MALAT1 by targeting several signaling pathways and miRNAs plays a pivotal role in CRC pathogenesis (Fig. 2).

**Table 1:** MALAT1-related signaling pathways in colorectal cancer (CRC)

MALAT1		Results	Refs.
Stimulate	Suppress		
WNT/β-catenin	–	Promote CRC cell invasion and metastasis	[69]
SFPQ PTBP2	–	Accelerate CRC cell growth and metastasis	[62]
AKAP-9	–	Stimulate CRC cell growth and invasion	[35]
Snail	−	Promote CRC cell EMT and migration	[56]
–	miR‑619‑5p	Increase the clinicopathological features of patients with CRC	[57]
EZH	E-cadherin miR-218	Enhance CRC cell EMT, metastasis, and chemoresistance	[22]
SOX9	miR-145	Promote CRC cell proliferation and migration	[64]
DCP1A	miR-203	Enhance CRC cell proliferation and invasion	[65]
HMGB1	miR‐129‐5p	Enhance CRC cell proliferation	[67]
ABC, BCRP, MDR1, MRP1	miR-20b-5p	Enhance CRC cell migration and reduce apoptosis and the sensitivity to drug	[70]
YAP1, VEGFA, SLUG, TWIST	miR-126-5p	Stimulate EMT and angiogenesis in CRC cells	[72]
LRP6/β-catenin, RUNX2	miR-15s	Enhance CRC cell metastasis	[73]
–	miR-194-5p	Enhance CRC cells migration and invasion	[74]
EZH2	miR-363-3p	Promote CRC cell proliferation	[58]
LC3-II	miR-101 p62/SQSTM1	Stimulate CRC cell proliferation and autophagy	[76]
–	miR-101-3p	Promote CRC cell viability	[78]
Wnt/β catenin, Bcl-2	Caspase-3, Bax	Enhance CRC cell proliferation and decrease apoptosis	[68]
ADAM17	miR-324-3p	Reduce the Ox-sensitivity in CRC cells	[82]
–	hsa-miR-194-5p	Decrease the PFS rate	[83]
RAB14	miR-508-5p	Promote CRC cell progression	[85]
IRE1/XBP1 and PERK/ATF4	–	Promote CRC cell migration and metastasis	[91]
FUT4 PI3K/AKT/mTOR	miR-26a/26b	Promote CRC cell invasion and tumorigenesis	[94]
lincRNA-ROR, lncRNA-p21, p53	–	Increase CRC cell tumorigenesis	[95]
DANCR	QK	Suppress apoptosis in CRC	[96]

MALAT1: Metastasis‐associated lung adenocarcinoma transcript‐1; AKAP-9: PRKA kinase anchor protein 9; CRC: Colorectal cancer; ABC: ATP-binding cassette transporters; BCRP: Breast cancer resistance protein; MDR: Multi-drug resistance proteins; YAP1: Yes-associated protein 1; DCP1A: mRNA‐decapping enzymes 1a; EZH2: Enhancer of Zeste 2; LC3-II/I: Microtubule-associated protein 1A/1B-light chain 3; SQSTM1: Sequestosome-1; ADAM17: A disintegrin and metalloprotease metallopeptidase domain 17; Ox: Oxaliplatin; PFS: Progression-free survival; PERK: Protein kinase R (PKR)‑like ER kinase; IRE1: Inositol‑requiring enzyme 1; ATF4: Transcription factor 4; XBP1: X‑box‑binding protein 1; QK: QUAKING; HMGB1: High motility group box protein 1; FUT4: Fucosyltransferase 4

Based on a previous study, MALAT1 as a prognostic risk factor decreased the survival outcomes of patients with CRC [54]. In advanced CRC patients, overexpression of MALAT1 is associated with drug resistance [22]. MALAT1 is able to increase oxymatrine resistance and the invasion ability of CRC cells [55]. MALAT1 by targeting at least 243 genes stimulates tumor development and enhances CRC cell invasion. The expression of PRKA kinase anchor protein 9 (AKAP-9) has been recognized that was increased in CRC tissues with lymph node metastasis [35].

A study reported that tumor-associated dendritic cells (TADCs) promoted migration and EMT in CRC [56]. C–C motif ligand 5 (CCL5) is a chemokine that mimics the impact of TADCs on CRC cells. Therefore, the inhibition of CCL5 expression via neutralizing antibodies or siRNA reduced cancer progression by TADCs. It has been suggested that Snail as the downstream target of MALAT1 participates in TADC-mediated CRC progression [56].

Further studies have found that MALAT1 can target miR‑619‑5p and increase the clinicopathological features of patients with CRC [57]. In CRC, MALAT1 through interaction with EZH2 can inhibit the expression of E-cadherin and induce Oxaliplatin (Ox) resistance. Also, MALAT1 interacts with miR-218 to enhance EMT, metastasis, and FOLFOX resistance [22]. MALAT1 by targeting miR-363-3p can enhance EZH2 expression levels and promote CRC cell proliferation [58].

PTBP2 or PTB (polypyrimidine-tract-binding protein) is a proto-oncogene that promotes the growth of CRC cells [59]. SFPQ or PSF is a PTB-associated splicing factor and a tumor suppressor gene that binds to PTBP2 [60]. MALAT1 has been observed that interacts with SFPQ, releases PTBP2 from the SFPQ/PTBP2 complex (SFPQ-detached PTBP2), and accelerates tumor growth and metastasis [61, 62].

Sex-determining region Y (SRY)-box 9 (SOX9) is a transcription factor that participates in CRC oncogenesis and metastasis [63]. MALAT1 by suppressing miR-145 could accelerate SOX9 mediated CRC cell proliferation, migration, and tumorigenesis (MALAT1/miR-145/SOX9 axis) [64].

MALAT1 has been proved that directly stimulates the expression of the mRNA‐decapping enzymes 1a (DCP1A), down-regulates miR-203, and enhances CRC cell proliferation and invasion (MALAT/miR-203/DCP1A axis) [65].

High mobility group box protein 1 (HMGB1) is a nuclear protein that enhances CRC cell development [66]. MALAT1 by targeting miR-129-5p increased the expression of HMGB1 (MALAT1/miR-129-5p/HMGB1 axis) and enhanced the proliferation of CRC cells [67].

Moreover, MALAT1 through the activation of Wnt/β-catenin signaling enhances CRC cell proliferation and reduces apoptosis (caspase-3 and Bax reduced, Bcl-2 increased) [68]. Resveratrol has been shown that down-regulates MALAT1 mediated the Wnt/β-catenin signal pathway and reduces CRC cell invasion and metastasis [69]. Therefore, the knockout of MALAT1 suppressed CRC cell migration and proliferation [54, 68].

MALAT1 by targeting key molecules participating in drug resistance, including breast cancer resistance protein (BCRP), ATP-binding cassette transporters (ABC), and multi-drug resistance proteins (MDR1 and MRP1) can increase the metastasis and invasion of CRC cells. Also, MALAT1 blocks the expression of miR-20b-5p and enhances CRC cell tumorigenesis. Hence, inhibition of MALAT1 reduced cell migration and promoted the sensitivity of CRC cells to 5-FU [70].

Yes-associated protein 1 (YAP1) has been reported that increases proliferation and migration of CRC cells [71]. YAP1 by targeting the MALAT1/miR-126-5p axis can stimulate vascular endothelial growth factor (VEGFA), SLUG, and TWIST as metastasis-associated molecules and control EMT and angiogenesis in CRC cells. miR-126-5p by blocking SLUG, TWIST, and VEGFA has a tumor suppressor role in CRC [72].

RUNX2 (Runt-related transcription factor 2) is a key transcription factor and proto-oncogene that plays an important role in various tumors. miR-15s by suppressing LRP6 expression (Wnt receptor) can block activation of β-catenin signaling. MALAT1 interacts with IRES domain in the 5′UTR of the RUNX2 mRNAs and increases translational levels of RUNX2. MALAT1 also via miR-15s/LRP6/β-catenin signaling positively regulates RUNX2 expression and enhances CRC cell metastasis [73].

MALAT1 was recently investigated that suppressed miR-194-5p and enhanced the migration and invasion of CRC cells [74]. In CRC tissues and cell lines, microtubule-associated protein 1A/1B-light chain 3 (LC3-II/I) reflects autophagosome formation [75]. There is a positive correlation between MALAT1 and LC3-II mRNA levels in CRC cells. MALAT1 by binding to miR-101 can stimulate CRC cell proliferation and LC3-II-induced autophagy, and suppress the expression of Sequestosome-1 (p62/SQSTM1) as an autophagosome cargo protein [76].

miR-101-3p was also reported to play as a tumor suppressor in various neoplasms [77]. A recent study confirmed that MALAT1 targeted miR-101-3p in radio-resistance cells and promoted CRC cell viability [78].

It has been found that high-dose Vitamin C administration has an inhibitory effect on MALAT1 and CRC metastasis [79].

A disintegrin and metalloprotease metallopeptidase domain 17 (ADAM17) is a protease for epidermal growth factor receptor (EGF-R) ligand processing [80]. It has been recently shown that ADAM17 can accelerate the tumorigenesis of CRC [81]. MALAT1 through suppression of miR-324-3p and stimulation of ADAM17 (as a target of miR-324-3p) could reduce the Ox-sensitivity of CRC cells in xenograft tumor mice treated with Ox [82]. Besides, MALAT1 was identified to inhibit the expression of the hsa-miR-194-5p and decrease the progression-free survival in patients with CRC [83].

RAB14 is a small GTPase member of the RAS oncogene family that enhances CRC cell proliferation [84]. MALAT1 as a ceRNA can target miR-508-5p and RAB14 (as a target of miR-508-5p) promote CRC progression [85].

Based on previous studies, endoplasmic reticulum (ER) stress through the unfolded protein response (UPR) pathway is contributed to CRC metastasis [86, 87]. It has been found that the protein kinase R (PKR)‑like ER kinase (PERK), inositol‑requiring enzyme 1 (IRE1), and transcription factor 6 (ATF6) activate signaling pathways involved in the UPR [88]. ER stress by suppressing cyclin D1 (cell cycle machinery) and inducing apoptosis plays an important role in CRC metastasis [89]. Thapsigargin (TG) is an ER stress inducer that stimulates cell migration [90]. TG‑induced MALAT1 is associated with the expression of the PERK and IRE1 pathways. Moreover, in CRC tissue samples, MALAT1 is positively regulated with the X‑box‑binding protein 1 (XBP1) and ATF4 binding sites. Therefore, the IRE1/XBP1 and PERK/ATF4 signaling pathways are involved in MALAT1-induced CRC progression [91].

Exosomes also play critical roles in the progression of CRC [92, 93]. A previous study showed that highly metastatic CRC-derived exosomes could accelerate the fucosyltransferase 4 (FUT4) levels (a key enzyme of fucosylation), invasion, and metastasis in primary CRC cells. They indicated that MALAT1 by targeting miR-26a/26b promoted FUT4-associated fucosylation, stimulated the PI3K/AKT/mTOR pathway, and increased CRC cell proliferation and tumorigenesis (MALAT1/miR-26a/26b/FUT4 axis) [94].

A study identified that MALAT1 can interact with lincRNA-ROR, lncRNA-p21, p53 and increase the tumorigenesis of CRC cells [95].

The RNA-binding protein QUAKING (QK) is involved in apoptosis and the RNA stability of MALAT1. Recently, DANCR (lncRNA) was found to mediate the interaction between QK and MALAT1 (DANCR/QK/MALAT1 axis), increase the anti-apoptotic function of MALAT1, and reduce Doxorubicin (Dox)-induced apoptosis in CRC cells [96].

Therefore, compared to traditional methods, MALAT1 can be a novel biomarker for the early diagnosis and prognosis of CRC.

## Conclusion

In this review, we highlighted the recently reported mechanism of MALAT1 in CRC. MALAT1 targets several signaling pathways such as Wnt/β-catenin, YAP, SOX9, RUNX2, Snail, EGF, PI3K/AKT/mTOR, and VEGF. Besides, MALAT1 has been found to modify miRNAs-associated drug sensitivity in CRC. Although these studies showed that MALAT1 plays a pivotal role in CRC tumorigenesis, the exact mechanisms whereby MALAT1 stimulates CRC development or invasion remains largely unclear. Taken together, the MALAT1-mediated treatment can be a critical therapeutic target for chemotherapy and radiotherapy sensitization.

## Data Availability

The datasets used and/or analyzed during the current study are available from the corresponding author on reasonable request.

## Abbreviations

ABC: ATP-binding cassette transporters
ADAM17: A disintegrin and metalloprotease metallopeptidase domain 17
AKAP-9: PRKA kinase anchor protein 9
ATF6: Transcription factor 6
BCRP: Breast cancer resistance protein
CCL5: C–C motif ligand 5
ceRNA: Competing endogenous RNA
CRC: Colorectal cancer
DCP1A: Decapping enzymes 1a
EMT: Epithelial-mesenchymal transition
ER: Endoplasmic reticulum
EZH2: Enhancer of Zeste 2
FUT4: Fucosyltransferase 4
HCN: Hepcarcin
HMGB1: High mobility group box protein 1
H3K27me3: Trimethylation of histone H3 at lysine 27
IBP160: Intron binding protein
IRE1: Inositol‑requiring enzyme 1
LC3-II/I: Microtubule-associated protein 1A/1B-light chain 3
LncRNA: Long non-coding RNAs
MALAT1: Metastasis‐associated lung adenocarcinoma transcript‐1
mascRNA: MALAT1-associated small cytoplasmic RNA
MDR: Multi-drug resistance proteins
miRNAs: MicroRNAs
NSCLC: Non-small cell lung cancer
NEAT2: Nuclear-enriched abundant transcript 2
Ox: Oxaliplatin
PERK: Protein kinase R (PKR)‑like ER kinase
PFS: Progression-free survival
PRC2: Polycomb repressive complex 2
PTB: Polypyrimidine-tract-binding protein
RNPS1: RNA-binding protein with serine-rich domain 1
RUNX2: Runt-related transcription factor 2
SQSTM1: Sequestosome-1
SRm160: Serine/arginine-rich (SR)-related protein
SRSF: SR splicing factor
TADCs: Tumor-associated dendritic cells
UPR: Unfolded protein response
VEGFA: Vascular endothelial growth factor
YAP1: Yes-associated protein 1
